# Preliminary Results from an RCT Examining the Effects of a Health Behavior Intervention as an Adjunct to Standard Trauma Therapy Among Adults with PTSD

**DOI:** 10.3390/brainsci15080871

**Published:** 2025-08-15

**Authors:** Jeffrey L. Kibler, Karla Patricia Molina Valenzuela, Shalynn Murphy, Claudia Ocholski, Dania Dabbagh, Valeria Rangel Cunha, Mindy Ma

**Affiliations:** College of Psychology, Nova Southeastern University, 3300 S. University Dr., Fort Lauderdale, FL 33328, USA

**Keywords:** PTSD, health behaviors, intervention, healthy lifestyle, physical activity, sleep

## Abstract

Background/Objectives: Individuals with posttraumatic stress disorder (PTSD) tend to show patterns of elevated cardiovascular disease (CVD) risk earlier in life than the general population. The need for effective interventions for CVD risk-reduction in PTSD is increasingly evident. In this paper we present preliminary results from a longitudinal study of a health behavior intervention, as an adjunct to standard trauma therapy in PTSD. The health behavior intervention addresses CVD-related heath behaviors (physical activity, nutrition, sleep, and stress) in a 12-week program delivered individually in 90-min sessions. Behavior change recommendations included: increased aerobic activity; establishing a balanced diet, enhancing consumption of fruits and vegetables and reducing sugars and fat/saturated fat; incorporating strategies to enhance sleep and lower PTSD-related disruptions (e.g., nightmares); and relaxation and cognitive coping skills to reduce general stress. Methods: Participants were randomized to the health behavior intervention plus standard trauma therapy experimental condition or a standard trauma therapy control group. Outcomes were measured at baseline and after the 12-week intervention phase. Sleep efficiency was measured from actigraphy watches. Physical activity was assessed by self-report and blood pressure was measured using an automated device. The preliminary outcomes are for 29 participants to date who have pre-post data. Results: Sleep efficiency was improved in the intervention group compared to controls (*p* < 0.05). The intervention group also evidenced significant pre-post increases in moderate physical activity compared to the control group (*p* < 0.05). Changes in vigorous physical activity did not reach statistical significance in this preliminary sample but the pattern of results are similar to those for moderate activity. Trends toward significance were also observed for pre-post changes in systolic (*p* = 0.06) and diastolic blood pressure (*p* = 0.07), with small reductions for the intervention group and increases for the control group. Conclusions: These findings provide preliminary information about the effectiveness of the health behavior intervention on multiple parameters for adults with PTSD. The findings suggest that focusing on health behavior change in multidisciplinary treatments for PTSD may enhance outcomes such as sleep and physical activity and potentially result in greater quality of life. However, the small preliminary sample size reported here should be considered when interpreting the outcomes. Further research may also determine how improvements in health parameters impact other indices of long-term cardiovascular health.

## 1. Introduction

Research has indicated strong associations between posttraumatic stress and cardiovascular disease (CVD) risk [1,2,3,4,5,6,7]. In comparison to the evidence concerning elevated CVD risk in PTSD, little research has addressed CVD risk-reduction in this population [3,8,9,10,11,12,13]. Thus, a strong need for developing effective interventions for CVD risk-reduction in PTSD is increasingly evident. In a study of Veterans treated in VA PTSD specialty clinics, those whose PTSD symptoms improved did not show significant improvement in CVD risks [13]. Adjunctive treatments, such as health behavior interventions, may be necessary as supplements to traditional psychotherapy for PTSD to reduce CVD risks. Health behavior interventions are needed that specifically address unique aspects of PTSD symptom presentation, which serve as barriers to healthy behaviors (e.g., avoidance of physiological arousal/activation, nightmares/sleep disruption, and cognitive responses to stress) and may not be adequately targeted with typical mental health treatments for PTSD [3,9,11,12,13].

Recurring trauma-related nightmares in PTSD, along with behavioral conditioning of sleep avoidance, are disruptive, prolonging sleep latency and contributing to irregular bedtimes [14,15]. Moreover, these types of disruptions in sleep have relevance for CVD risk/outcomes [13,16,17,18]. Scherrer et al. [13] found that sleep disorders mediated associations of PTSD with CVD. Thus, sleep interventions may enhance CVD risk-reduction efforts [18,19].

Typically, psychotherapies for PTSD do not address sleep problems directly or systematically; further, sleep outcomes following therapy have not generally been assessed [20,21]. The limited number of cognitive behavioral therapy (CBT) interventions that have focused specifically on sleep in PTSD have resulted in improved sleep [22,23]. Thus, further research in this area of treatment may assist in evaluating the potential for health behavior intervention to impact health in PTSD.

Fear of bodily arousal symptoms (e.g., shortness of breath, racing heart rate) has been associated with less exercise and lower ratings of exercise motivation in PTSD [24,25,26]. In conjunction with anxiety reactions, the fear of bodily arousal can be conceptualized as anxiety sensitivity, which is commonly reported among those with PTSD [24,27,28].

Anxiety sensitivity may lead to avoidance of exercise [29]. However, evidence suggests that engaging in exercise can decrease anxiety sensitivity [24,25,30], potentially reversing the cycle of arousal avoidance. These effects are theorized to occur through exposure and desensitization to internal arousal cues [30]. The potential for fear of arousal suggests the need to begin slowly when implementing exercise for individuals with PTSD who are not active. Strong theoretical and empirical evidence indicates that abrupt increases in activity are not as likely to be maintained [31]. Abrupt changes in exercise may be more difficult when one has fear or sensitivity to arousal [31]. Therefore, our experimental intervention was designed to be sensitive to the specific needs of participants with PTSD (i.e., to allow participants to work at their own pace). Hall et al. [32] found that increasing physical activity is feasible and can lead to better aerobic performance and metabolic function in older military Veterans with PTSD.

The purpose of the present paper is to present the preliminary results from our longitudinal study of a health behavior intervention, as an adjunct to standard trauma therapy in PTSD. The health behavior intervention consists of four overlapping modules focused generally on physical activity, nutrition, sleep, and stress management (the specifics are detailed in the Method section). It was hypothesized that the healthy lifestyle intervention would result in greater improvement in cardiovascular risks, as reflected in three of our primary outcome measures (sleep efficiency, physical activity, and blood pressure), beyond the effects of standard trauma therapy (control group).

## 2. Method

### 2.1. Participants

The present paper includes data from participants who have completed the baseline and post assessments to date (*N* = 29), with 18 from the experimental health behavior intervention group and 11 from the control group. The demographics from this sample are age 30 ± 11 (mean ± SD) and 96% female (1 male). Race/ethnicity was reported as 52% Hispanic-White, 7% Hispanic Black, 24% non-Hispanic White; 10% non-Hispanic Black, and 7% Asian. For educational attainment, 10% of the sample reported high school as the highest degree earned, 28% reported education to a bachelor’s degree, and another 21% reported a Master’s and/or doctoral or professional degree; however, a significant proportion of participants (41%) responded “other” or did not answer the item regarding education.

Women and men ages 18+ were recruited from the community and local clinics. Study candidates self-referred by calling a private study number or responding to a dedicated study email address.; they were screened by phone to assess study inclusion/exclusion criteria, and were also given additional details about the study. Criteria for inclusion were: PTSD or subthreshold PTSD [evidenced by ≥4 PTSD symptoms (including ≥1 re-experiencing symptom) endorsed as 2 or higher (moderately bothersome) on the Posttraumatic Stress Checklist (PCL-5)], reporting at least one health risk (low physical activity, nutrition does not meet standard recommendations, overweight, sleep difficulties), and ability to exercise at a moderate level. Concurrent trauma-focused psychotherapy was required through the 12-week pre to post intervention phase (at least twice per month) and was provided by the project if the participant was not already receiving standard trauma-focused therapy in the community. On an ongoing basis, the project coordinator verified the therapy and frequency. Although study candidates were not excluded based on chronic illnesses, medications to control blood pressure were exclusionary. Comorbid mental health conditions (e.g., depression, personality disorders, substance use disorders) were not exclusionary because this would result in an unnatural restriction among the adult sample with PTSD. Those on psychopharmacological medications were included if the regimen had been maintained as stable for ≥two months prior to enrollment. This study is approved by the appropriate University IRB. Written informed consent was obtained from all participants at the time of the first outcome assessment (baseline) by the laboratory research assistant. Referrals for continued care/therapy beyond the study intervention phase are provided by the study team.

Randomization to study condition is performed by the project coordinator following the initial phone contact (using the flip of a coin). Neither the participants nor the investigators are blinded for this trial. Limitations of both participant and research team awareness of study condition for each participant could include demand characteristics displayed due to expectations about the study and the experimental intervention. This could impact behaviors and in turn potentially impact health-related outcomes. However, given the nature of the study (participation in an active behavioral intervention) and level of involvement of advanced research coordinators in multiple study domains, we determined that it would not be possible to effectively blind the participants or study team to the conditions while still providing participants adequate information about the study design at consent. Efforts to mitigate bias include restricting feedback and sharing of results/outcomes with participants until the entire study protocol is complete to minimize response biases.

The baseline assessment session included additional screening assessments to evaluate eligibility based on PTSD symptoms and the presence of at least one risk factor targeted by the intervention (e.g., physical inactivity, sleep difficulties).

### 2.2. Measures

Outcome Assessment Sessions. The pre- and post-intervention assessment sessions were conducted in person at a University-based laboratory. The first assessment (baseline) was conducted prior to the first week/study onset in each condition. The second assessment (post-treatment) was conducted at the 3-month time point (after the last healthy lifestyle session in the experimental condition). Each assessment session consisted of the same outcome measures (e.g., blood pressure, physical activity). Due to potential confounding effects on blood pressure, participants are asked to refrain from caffeine and strenuous exercise for 3 h, and smoking for 30 min, prior to assessment sessions. Noncompliance with restrictions required rescheduling. A doctoral research assistant conducted each assessment session. The data collection for the assessment included the following:

Blood Pressure. The participant sat quietly for 5 min, after which three seated, automated systolic blood pressure (SBP) and diastolic blood pressure (DBP) readings (IntelliSense model 5243175; Omron Healthcare Inc., Kyoto, Japan) were taken at 2-min intervals.

An online computer assessment was utilized for the assessment of physical activity, demographic information, and medication use:

Physical Activity. The International Physical Activity Questionnaire (IPAQ; 7-day long form), a 27-item measure, developed through international collaboration to be a standard for physical activity assessment, was used to assess time spent in moderate and vigorous activity over the past week, aligning with current activity guidelines [33,34]. The IPAQ demonstrates strong test–retest reliability (ICC = 0.66–0.88) and moderate validity, with correlations of *r* = 0.30–0.50 with accelerometer data [35,36]. The IPAQ’s standardized format ensures consistent and valid assessment of physical activity patterns across diverse populations.

Demographic Measures. Basic demographic information, including age, race, ethnicity, family income, marital status, and education level, was assessed by self-report.

Medication Use. Medication use was assessed by self-report during the screening period to determine participant eligibility, ensuring that current medications do not confound study outcomes. At each lab assessment (pre and post), participants were asked to report any changes in medication use. An open-response option was provided to capture detailed information, allowing participants to type in the names and dosages of their specific medications.

Accelerometry. Participants were provided with an Actigraph wGT3X-BT watch (Ametris, Pensacola, FL, USA) and instructed on its use to track the accelerometer-based sleep measures each day for one week following each assessment session. After the watch was returned, the data were downloaded for analysis. The wGT3X-BT is the industry standard for accelerometer monitoring of physical activity and sleep [37,38]. The GT3X+ device is small (weighing only 19 g), unobtrusive, tamper-resistant, well-tolerated, and does not hinder activity [37]. The accompanying ActiLife software version 6.13.6 was utilized for data download and analysis.

### 2.3. Procedures

Study Interventionists and Training. The interventionists were advanced doctoral trainees with experience delivering CBT informed health interventions and/or trauma-focused therapies (depending on the protocols they are trained to conduct—health behavior program and/or standard trauma therapy). The health behavior interventionists had prior exposure to the healthy lifestyle intervention literature and furthered their understanding of the protocol for PTSD patients by reviewing the manual and meeting with the first author (Dr. Kibler, study principal investigator) for training. The number of doctoral assistants selected to provide the health behavior intervention was limited in order to maintain as much consistency as possible. Interventionists met with Dr. Kibler through the intervention delivery to address any questions that might relate to participant-specific presentations, and implementation of the health behavior sessions was also discussed in team meetings to further enhance consistency and intervention quality. Participants had the option of conducting intervention sessions (health behavior and/or trauma therapy sessions) in-person or virtually via a secure HIPAA-compliant Zoom platform.

Interventionists who conducted the standard trauma therapies (delivered individually to participants) reviewed evidence-based conceptual and intervention models of trauma-focused treatment for PTSD, including Cognitive Processing Therapy [39,40], Prolonged Exposure Therapy [41,42], and, as applicable for participants with a presentation consistent with the literature on complex-PTSD, Contextual Trauma Therapy [43]. Trauma therapists completed treatment-specific readings for the various models of treatment reviewed during training and met with Dr. Kibler and project coordinators (designated as lead interventionists) to discuss treatment options for assigned participants. Trauma therapists select treatment approaches based on the clinical presentations of participants and collaboration with participants about the options for care.

Health Behavior Intervention. The health behavior intervention is a 12-session program developed by the first author and delivered individually to participants with four modules for increasing physical activity, and improving nutrition, sleep, and stress management. The sessions are delivered individually, once per week for 90 min.

Physical activity is the focus of the first module, including discussion of health benefits of physical activity and health risks associated with inactivity and overweight. The interventionist and participant also discuss implementation of an exercise regimen and potential challenges/barriers. Starting in session 1, participants are encouraged to begin or maintain physical activity that is consistent with standard guidelines (aerobic exercise 2–3 times per week for at least 20 min). However, interventionists engage participants in working on exercise goals that can be reasonably incorporated into their schedule and maintained. Participants also walk for 20 min with the interventionist in sessions 2–5. Session 2 also includes discussion of healthy food choices, using the MyPlate food groups, health risks associated with unhealthy foods, appropriate caloric intake for weight loss, reading food labels, fat, vitamin, and sugar content, and setting dietary goals. The interventionist helps the participant distinguish diets from lifestyle changes, emphasize self-monitoring of foods, and build strategies for nutritional self-control. Nutritional recommendations include a balanced diet, enhancing consumption of fruits and vegetables, and reducing sugars, fat/saturated fat, and caloric intake (where appropriate). During sessions 3 and 4, the participant and interventionist work together to analyze current nutritional habits and plan a healthier nutritional plan. Session 3 includes discussion of the participant’s efforts to incorporate physical activity and healthy nutrition into their lifestyle, and the potential for social support to be helpful in behavior change. This session also addresses stress and eating. Interventionists closely monitor the stress coping skills of participants and discuss potential distress associated with giving up previously used unhealthy eating approaches to cope (especially in coping with PTSD symptoms). To address the potential desire to be overweight for protection against abuse, alternative methods of achieving a sense of safety are discussed. Session 4 focuses on the skills of shopping for healthy food, planning healthy meals, and reducing behavioral cues/triggers for binge eating and unhealthy eating (e.g., storing foods out of sight, strategies for parties, special events and restaurants, managing emotions).

Sleep interventions are implemented in sessions 5–8. In session 5, an overview of common sleep problems is presented, and the participant reviews any of their own sleep difficulties. The interventionist also discusses the rationale for the treatment used in our protocol. Sessions 6–8 include presentation and practice of cognitive behavioral strategies for improving sleep. Participants also identify a problematic nightmare (where applicable) during session 6, and begin the imagery rehearsal therapy protocol by beginning to write a modified version of the dream. Insomnia intervention strategies are discussed in sessions 7 and 8, as well as having participants further develop their modified dream and rehearse it in session. In addition, participants are asked to rehearse the modified dream daily, outside of the sessions, for 5–20 min.

Stress management is covered in the final sessions (9–12), along with relapse prevention and other discussion relevant to termination of the program. The interventionist provides an overview of the cognitive behavioral model of stress during session 9, and also engages the participant in discussing various aspects of the model (i.e., the path from negative appraisals to emotional and behavioral reactions). For homework after session 9, the participant is instructed to self-monitor negative thoughts. This assignment facilitates follow-up discussion during session 10. The focus of session 10 is on patterns of negative appraisals and negative emotions that may result; participants are also introduced to thought logs [44], which use personal situations to assist participants in recognizing associations between negative appraisals, negative emotions, and behavioral reactions. Conversely, more adaptive approaches to responding in stressful situations are also discussed. For homework between session 10 and 11, participants complete two or more thought logs. The material from the thought logs is processed in session 11. Review of thought logs and stress coping is continued in session 12 for the first part of the session; the remainder of the final session includes a review of accomplishments, discussion of higher risk situations for relapse, using skills for relapse prevention, and any remaining concerns associated with finishing the program.

### 2.4. Data Reduction and Statistical Analyses

Statistical analyses were conducted using SPSS software version 29. Preliminary analyses were conducted to examine distributions, detect outliers, and compare the study groups on demographic variables. Table 1 depicts the demographic variables by condition; the groups were not significantly different on any of the demographic parameters.

A between-groups statistical approach was used, with study condition as the independent variable and changes in the outcomes as the dependent variables. The changes in outcome variables were analyzed with standard two-tailed t-tests. Effect sizes (Cohen’s *d*) were specified to provide additional information in the evaluation of outcomes, and to permit interpretation of small samples size in relation to power in analyzing statistical significance. The basic assumptions for t-tests were generally met (i.e., normality, sample size, homogeneity of variances). However, if the basic assumption of homogeneity of variances was not met for a t-test, the evaluation of significance was adjusted for unequal variances. There were no adjustments or controls for covariates or confounds in the analyses presented for this paper. For participants who were missing data for one of the outcomes, their data was not included in the respective analyses. No imputation or substitutions were implemented for the present paper.

## 3. Results

Results for actigraphy-based sleep efficiency analyses indicated a small improvement in pre-intervention (baseline) to post-intervention change in sleep efficiency for the intervention group (+0.87% improvement ± 2.5), that was statistically significant (*t* = 2.051; *p* < 0.05; effect size (*d*) = 0.86) compared with a reduction in sleep efficiency for the control group (−1.6% ± 3.2). Table 2 depicts the data from the pre and post assessments for each condition and the test of significance for changes in each outcome.

**Figure 1:**
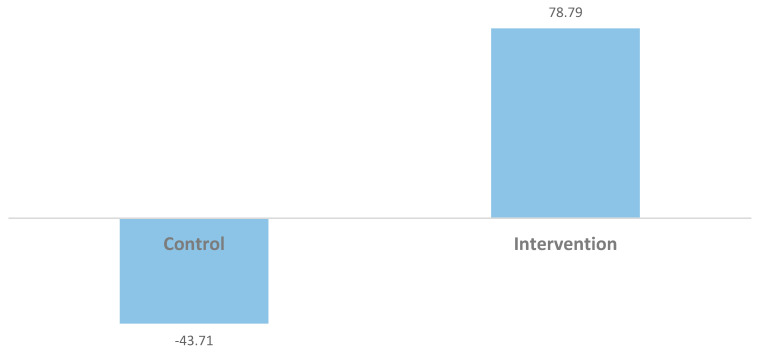
Mean difference in values (minutes) representing changes in moderate physical activity in a typical week from pre-intervention to post-intervention in the two study conditions.

The intervention group also evidenced significant pre-intervention to post-intervention increases in moderate physical activity compared with the control group (*t* = 2.817; *p* < 0.05; *d* = 1.03); mean changes in moderate physical activity are illustrated in Figure 1. Changes in vigorous physical activity did not reach statistical significance in this preliminary sample, but the pattern of results were similar to those for moderate activity [mean increase of 44.6 min of vigorous activity (in a typical week) for the intervention group compared with 9.2 min in the control group]; the effect size for this difference in physical activity was small to moderate (*d* = 0.31).

Results of analyses for blood pressure did not reach significance in this preliminary sample; although significant differences were not observed, the evaluations of these outcomes approached significance for pre-intervention to post-intervention changes in both systolic blood pressure (*t* = 1.843; *p* = 0.06; *d* = 0.68) and diastolic blood pressure (*t* = 1.983; *p* = 0.07; *d* = 0.79), with small reductions in blood pressure for the intervention group and increases for the control group (see Table 2).

## 4. Discussion

The preliminary data presented here support the hypothesis that the health behavior intervention program would have a significant impact on sleep efficiency and physical activity. Large effect sizes for most of our preliminary findings that were statistically significant, despite a relatively small sample for these analyses, suggest that these effects may be robust findings that are likely to persist in a larger sample. Given prior research indicating sleep disruption and lower physical activity in PTSD [14,26], the outcomes in the present study represent major changes that would enhance health and quality of life if changes are maintained for individuals with PTSD. Our plan for future examinations with the present study data is to evaluate the maintenance of changes over a 9-month follow-up period; assessments are made 3 months and 9 months after the post-assessment.

Our findings are consistent with the limited number of studies indicating physical activity interventions can be beneficial for individuals with PTSD [32,45]. We attribute the increases in moderate physical activity in the health behavior intervention group (at least in part) to enabling participants to work at their own pace and encouraging manageable increases in activity, while also addressing any fears of bodily arousal symptoms either in real time or on a weekly basis [24,25,30].

Our positive findings related to sleep efficiency suggest that our approaches involving cognitive-behavioral intervention for sleep hygiene and nightmares were successful in the short-term format of the sleep module [20,23]; this is notable given that previous reports have addressed these problems in a longer protocol for adults with PTSD [22,23]. Improving sleep efficiency has strong implications for cardiovascular health in PTSD [46]. The sleep disruption that is common in PTSD has been shown to impact several aspects of CVD risk [13,16,17,18,47]. In particular, actigraphy-derived sleep efficiency was recently shown to be associated with endothelial function and arterial stiffness in young trauma exposed women [46]. Intervening with sleep and enhancing sleep efficiency has the potential to improve these cardiovascular risk pathways for adults who are affected by PTSD, especially when interventions are implemented to prevent CVD risks at a relatively early age [46,47].

Overlapping modules in the health behavior program encourage building skills for a healthier lifestyle early in the intervention. In addition, focusing on multiple health behaviors at once may permit participants’ detection of interacting variables (e.g., a participant does not sleep well on days that they do not exercise) [48,49]. Following the focus on physical activity and nutrition in sessions 1–5, participants are instructed to continue implementing their physical activity and nutritional plans throughout the intervention, and ideally beyond completion of the program. Our efforts to address sleep in conjunction with other health behaviors may also confer a synergistic effect that is greater than the impact of each component alone [49,50]. One study showed a positive association between exercise intervention and improved sleep for individuals with PTSD [45].

The large effects for improvement in blood pressure suggest the possibility that these findings could be statistically significant with a larger sample, and that behavioral changes resulting from the health intervention may be impacting physiological outcomes. Each component of the health behavior intervention (physical activity, nutrition, sleep, and stress management) has the potential to impact reductions in blood pressure; further research will help us to establish the mechanisms associated with blood pressure improvements in our participants.

Some limitations of the present findings should be considered. As a preliminary examination of the findings, the sample size is small. The sample size could have limited the ability to detect significance for all the primary outcomes. A larger sample would also provide more firm conclusions about the consistency and generalizability of the findings. With a smaller sample the generalizability may not capture a wide enough range of participant characteristics. Another limitation is the potential for bias related to not blinding the participants and study team to the study conditions. For example, reporting of outcome data from participants in the experimental health behavior intervention or control group could be influenced by their expectations about the study.

There are several methodological strengths of the present study that enhance our evaluation of changes in health risks. The use of objective sleep monitoring using the ActiGraph technology provides a stronger basis for evaluating the changes in these parameters than self-report alone [51]. In addition, comparison of the experimental health behavior intervention plus standard therapy with a standard therapy control is an advantage in evaluating whether health benefits may be specifically related to the health behavior intervention (as opposed to general improvements in mental health).

## 5. Conclusions

Overall, our preliminary findings provide necessary data to evaluate whether a health behavior program can produce consistent improvements in health risk factors, which exceeds standard care for adults with PTSD. As treatment with standard psychotherapy for mental health symptoms in PTSD may not be sufficient to also confer improvement in CVD risks [13], health behavior interventions such as our experimental intervention may provide incremental value for intervening in CVD-related health behaviors. As is critical, the presently tested intervention addresses unique aspects of PTSD that serve as barriers to healthy behaviors (e.g., avoidance of physiological arousal, nightmares) and may not be adequately targeted with typical treatments for PTSD [3,11,12,13].

In conclusion, the present study includes an evaluation of several cardiovascular health risk outcomes following health behavior intervention in adults with PTSD. It provides a valuable assessment of the effects of a multi-component health behavior intervention designed specifically for this population. The study results inform multidisciplinary and/or multicomponent intervention that may have a significant impact on quality of life for patients with PTSD.

## Figures and Tables

**Table 1:** Pre-post sociodemographic data by participant group (experimental health behavior intervention vs. control group).

	Participant Group		Test of Significance
Variable	Health Behavior Intervention (*n* = 18)	Control Group (*n* = 11)	*p* Value
Mean Age (SD)	31.9 (10.6)	25.8 (9.6)	*p* = 0.15
Race/Ethnicity (# per group):			*p* = 0.72
Hispanic White	9	6	
Non-Hispanic White	5	2	
Hispanic Black	1	1	
African American	2	1	
Asian	1	1	
Education (# per group):			*p* = 0.44
High school or equivalent	2	1	
Associate’s Degree	0	0	
Bachelor’s Degree	6	2	
Master’s Degree	4	1	
Medical/Professional/Doctorate	0	1	
Other	6	6	
Family Income (# per group)			*p* = 0.27
<$5000	0	0	
$5000–$10,000	1	1	
$10,001–$15,000	1	0	
$15,001–$20,000	1	1	
$20,001–$30,000	2	0	
$30,001–$40,000	3	2	
$40,001–$50,000	4	2	
$50,001–$75,000	3	2	
$75,001–$100,000	2	0	
$100,000+	1	3	

**Table 2:** Pre-post outcomes by participant group (experimental health behavior intervention vs. control group).

	Participant Group				Test of Significance
Variable	Health Behavior Intervention (*n* = 18)		Control Group (*n* = 11)		*p* Value
	Pre/Baseline	Post	Pre/Baseline	Post	
Sleep Efficiency % (SD)	91.6 (4.5)	92.5 (3.4)	93.7 (4.4)	92.1 (2.4)	*p* < 0.05
Moderate Physical Activity in minutes per week (SD)	133.7 (145.1)	212.5 (214.5)	163.2 (55.0)	119.5 (125.5)	*p* < 0.05
Vigorous Physical Activity in minutes per week (SD)	16.3 (80.3)	60.9 (126.5)	58.5 (35.5)	67.7 (108.5)	*p* = 0.51
Systolic Blood Pressure (SD)	111.1 (14.1)	109.6 (11.8)	100.2 (11.3)	103.9 (9.5)	*p* = 0.07
Diastolic Blood Pressure (SD)	75.6 (9.1)	73.9 (8.4)	67.0 (5.7)	70.6 (7.9)	*p* = 0.06

## Data Availability

The datasets presented in this article are not readily available because the data are part of an ongoing study. Requests to access the datasets should be directed to the first author (Dr. Kibler). The project PI (Dr. Kibler) will share final data from the project when the criteria outlined below have been met, and after data collection is complete. In such circumstances, data will be shared directly with the requestor, taking appropriate precautions to protect participant confidentiality. Data will only be shared that are not part of the primary data analysis plan of the project, and the PI reserves the right to develop secondary analysis plans that would limit the availability to requestors. The PI may also request to work collaboratively with requestors to develop the data into publications and presentations if there are topics of mutual interest. Those requesting access to the project data must submit a request in writing to the PI, which addresses participant protection (IRB procedures, protection of participant confidentiality/identifiers), the scope of the proposed work, and the timeframe for the proposed research and deliverables (dissemination of the work). In the event that approval of data sharing is provided, only the requested data/variables will be provided to the requestor.
